# Sonic Hedgehog Regulates Proliferation, Migration and Invasion of Synoviocytes in Rheumatoid Arthritis *via* JNK Signaling

**DOI:** 10.3389/fimmu.2020.01300

**Published:** 2020-06-24

**Authors:** Shangling Zhu, Yuanmei Ye, Yiming Shi, Junlong Dang, Xiaoxue Feng, Yingdi Chen, Fang Liu, Nancy Olsen, Jianlin Huang, Song Guo Zheng

**Affiliations:** ^1^Department of Rheumatology, The Sixth Affiliated Hospital of Sun Yat-sen University, Guangzhou, China; ^2^Department of Internal Care Unit, The Sixth Affiliated Hospital of Sun Yat-sen University, Guangzhou, China; ^3^Department of Clinical Immunology, The Third Affiliated Hospital of Sun Yat-sen University, Guangzhou, China; ^4^Department of Medicine, Penn State Hershey Medical Center, Hershey, PA, United States; ^5^Department of Internal Medicine, Ohio State University College of Medicine and Wexner Medical Center, Columbus, OH, United States

**Keywords:** rheumatoid arthritis, fibroblast-like synoviocyte, proliferation, migration, invasion, sonic hedgehog, c-Jun N-terminal kinase

## Abstract

Activated fibroblast-like synoviocytes (FLSs) play a central role in the formation of synovial pannus and joint destruction in rheumatoid arthritis (RA). Targeting FLSs could be a potential therapeutic strategy. The objective of this study is to explore the role of c-Jun N-terminal kinase (JNK) in proliferation, migration and invasion of FLSs promoted by the sonic hedeghog (SHH) signaling pathway in patients with RA. Activation of SHH signaling was evaluated by real-time PCR and Western Blot. Levels of phosphorylation of JNK and c-Jun were detected by Western Blot. FLSs proliferation was quantified by Cell Counting Kit-8 (CCK-8) assay and flow cytometry. Cell migration and invasion were assessed by wound healing assay and Transwell chamber assay. Invasiveness of FLSs *in vivo* was evaluated using a humanized synovitis animal model. We observed that treatment of SHH agonist (SAG) significantly increased the levels of phosphorylation of JNK and c-Jun, while SHH antagonist (cyclopamine) significantly decreased the expression of phospho-JNK and phospho-c-Jun in FLSs. The elevated level of phospho-c-Jun stimulated by SAG was decreased in the presence of JNK inhibitor (SP600125) (*P* < 0.001). FLSs proliferation, migration and invasion were promoted by SHH agonist (*P* < 0.05). However, the enhanced aggressiveness of FLSs was abolished in the presence of JNK inhibitor (*P* < 0.05). *In vivo* study showed that the invasion of FLSs into cartilage was increased by SHH overexpression and the excessive invasiveness was inhibited by blockade of JNK signaling (*P* < 0.01). These results suggest that JNK is one of the downstream molecules mediating the effect of SHH signaling in FLSs. These findings indicate that SHH-JNK signaling could be a potential therapeutic target to suppress the aggressiveness of FLSs and prevent articular damage of RA.

## Introduction

Rheumatoid arthritis (RA) is an immune-mediated inflammatory disease characterized by inflammation and damage in the joints (1). Activated fibroblast-like synoviocytes (FLSs), the major cell population in the hyperplastic synovial intimal lining, have a central role in the formation of synovial pannus and joint destruction (2). In RA, FLSs interact with immune cells and produce abundant inflammatory cytokines and chemokines, contributing to the perpetuation of inflammation (3–8). In addition, FLSs derived from RA patients display an aggressive phenotype of excessive proliferation, resistance to apoptosis and enhanced capacities to migrate and invade, the latter considered as the main cause of tissue damage (9). Therefore, understanding underlying mechanism(s) of aggressive features of FLSs has the potential to find novel targets for the treatment of RA, which may reduce inflammation as well as bone erosion (10–12).

The sonic hedgehog (SHH) signaling pathway is a conserved pathway and plays a critical role in embryonic development, stem cell maintenance and tissue homeostasis. SHH signaling is tightly regulated by various molecules. Briefly, the SHH ligand binds to the membrane receptor Patched1 and activates the G-protein coupled receptor-like protein named Smoothened (SMO). Subsequently, downstream molecules of SMO facilitate the signaling transduction from cytoplasm to nucleus, leading to the activation of the transcriptional factors GLI family zinc finger 1–3 (GLI1-3). Among the three GLI factors, GLI1 functions as a transcriptional activator and a target gene, while GLI2 and GLI3 are the mediators of signaling transduction and can act as activators or repressors according to the cellular context (13). Suppressor of Fused (SUFU) can directly bind to GLI transcription factors and act as a major negative regulator by promoting the generation of the repressor form of GLI or by preventing translocation to the nucleus (14, 15).

Inappropriate activation of SHH signaling was implicated in the tumorigenesis and metastasis of various human tumors. The underlying molecular mechanism is controversial; however, activated SHH signaling is proven to contribute to increased cell proliferation, differentiation and epithelial-to-mesenchymal transition which is correlated with cell invasion and metastatic abilities of tumors (16, 17). We have previously reported that SHH signaling was activated in the synovial tissue of RA patients (18). In addition, SHH signaling promoted FLSs proliferation and migration (19, 20), indicating that SHH signaling contributes to the aggressive behavior of FLSs. However, the mechanism by which SHH signaling promotes the tumor-like behavior of FLSs is still not yet fully elucidated.

Increasing evidence has suggested that mitogen-activated protein kinase (MAPK) signaling, including extracellular signal-regulated kinase (ERK), c-Jun N-terminal kinase (JNK), and p38, is implicated in the inflammation and joint destruction of RA (21, 22). We have recently reported that SHH signaling promoted FLSs proliferation and migration through activating ERK/MAPK (23). Therefore, we sought to determine the role of other MAPK signaling in SHH-induced FLSs proliferation, migration and invasion in the current study.

## Materials and Methods

### Ethics and Samples

The study was approved by the Medical Ethics Committee of the Sixth Affiliated Hospital of Sun Yat-sen University. All patients provided written informed consent to participate in the study.

Nine patients with active RA, including 2 males and 7 females (aged from 46–59 years) were recruited, and synovial tissue samples were obtained during knee arthroscopy or synovectomy. RA patients were classified according to the 1987 American College of Rheumatology (ACR) revised classification criteria (24) and exhibited moderate to severe disease activity (Disease Activity Score of 28 joint counts >3.2). Normal cartilages were obtained from patients with traumatic injuries who were undergoing hip surgery. Animal study was approved by Guangdong Laboratory Animals Monitoring Institute.

### Cell Culture

The synovial tissue biopsies isolated from RA patients were finely minced into small pieces and transferred to a tissue culture flask in Dulbecco's modified Eagle's medium (DMEM) (Hyclone Laboratories, LOGAN, UT, USA) supplemented with 10% fetal bovine serum (FBS) (Gibco, Grand Island, NY, USA), 100 U/mL penicillin (Gibco) and 100 μg/mL streptomycin (Gibco) in a humidified incubator at 37°C under 5% CO_2_. FLSs usually migrated out from the tissue and grew in monolayer in 10–14 days. After growth to ~95% confluency, cells were trypsinized and planted for proliferation. FLSs from passages 3–5 were used for planned experiments after being confirmed as being FLSs by morphology and purity analysis (<1% CD11b-positive, <1% CD 68-positive, <1% FcgRII and FcgRIII receptor-positive and >96% CD90-positive) (Supplementary Figure 1).

### Quantitative Real-Time PCR

FLSs were treated with SHH agonist SAG (1 μM, Calbiochem, Darmstadt, Germany) or antagonist cyclopamine (10 μM, Sigma-Aldrich, Saint Louis, MO, USA) for 24 h. For the examination of production of matrix metalloproteinases (MMPs) and expression of IL-6 and IL-8, FLSs were treated with SAG (1 μM) alone or SAG with JNK inhibitor SP600125 (20 μM, Sigma-Aldrich, Saint Louis, MO, USA) for 24 h.

Total RNA from FLSs was prepared using E.Z.N.A. ®Total RNA Kit (OMEGA, Omega Bio-tek, Norcross, GA, USA) according to the manufacturer's protocol. cDNAs were synthesized using the PrimeScript™ RT reagent Kit with gDNA Eraser (Takara Biotechnology, Dalian, China) according to the manufacturer's instructions. To quantify the relative expressions of target genes, real time PCR was performed using TB Green® *Premix Ex Taq*™ II Kit (Takara Biotechnology) on a QuantStudio 5 Real-Time PCR System (Thermo Fisher Scientific Inc.,Waltham, MA, USA). All experiments were performed in triplicate and repeated at least three times with similar results. Relative levels were quantified by the comparative delta Ct method.

Primers for amplification were as follows (forward, reverse): SMO: (CCTGCTCACCTGGTCACTC, CACGGTATCGGTAGTTCTTGTAG), SUFU: (GCACATGCTGCTGACAGAGGAC, AGACACCAACGATCTGGAGGAAGG), GLI1: (AGGGAGTGCAGCCAATACAG, CCGGAGTTGATGTAGCTGGT), MMP1: (TTGCAGCTCATGAACTCGGC, CCGATGGGCTGGACAGGATT), MMP13: (TCCTGGCTGCCTTCCTCTTCTTG, AGTCATGGAGCTTGCTGCATTCTC), IL-6: (GGTGTTGCCTGCTGCCTTCC, GTTCTGAAGAGGTGAGTGGCTGTC), IL-8: (TCTCTTGGCAGCCTTCCTGA, TTTCTGTGTTGGCGCAGTGT), β-actin: (GGACTTCGAGCAAGAGATGG, AGCACTGTGTTGGCGTACAG).

### Western Blot Analysis

To further verify the effects of SMO agonist and antagonist on SHH signaling, the protein expression of SMO, SUFU, and GLI1 was determined by Western Blot analysis. The activation of JNK signaling was measured by quantifying phosphorylation of JNK and c-Jun after SAG or cyclopamine treatment. For the detection of MMP1 and MMP 13, FLSs were treated with SAG (1 μM) alone or SAG with JNK inhibitor SP600125 (20 μM) for 48 h. Briefly, total protein was extracted using RIPA buffer (Sigma, Shanghai, China) supplemented with a cocktail of protease inhibitor (Roche, Mannheim, Germany), phosphatase inhibitor (Roche, Mannheim, Germany) and Phenylmethanesulfonyl fluoride (Sigma, Shanghai, China). For the detection of GLI1, nuclear protein was isolated using NE-PER Nuclear and Cytoplasmic Extraction Reagents (ThermoFisher Scientific, Rockford, IL, USA) according to the manufacturer's instructions. Polyacrylamide gel electrophoresis (PAGE) was performed to separate the proteins using 4–12% SurePAGE™ Gels (GeneScript, Piscataway, NJ, USA). The proteins were blotted onto a polyvinylidene fluoride (PVDF) membrane. After blocking at room temperature for 1 h, the membranes were incubated overnight at 4°C with primary antibodies. Primary antibodies were diluted 1:1000 for anti-SMO (Abcam, Cambridge, UK), anti-SUFU (Cell Signaling Technology, Shanghai, China) and anti-GLI1 antibody (Abcam). For the detection of JNK signaling, primary antibodies including anti-phospho-JNK, anti-JNK, anti-phospho-c-Jun and anti-c-Jun antibodies (1:1000, Cell Signaling Technology) were used. To detect the expression of MMP1 and MMP13, anti-MMP1 and anti-MMP13 antibody (1:1000, Abcam) were used. Subsequently membranes were incubated for 1 h at room temperature with secondary antibodies (1:5000, Cell Signaling Technology) conjugated with horseradish peroxidase. Immobilized proteins were measured by the enhanced chemiluminescent (ECL) detection system. The quantification of band density was performed using ImageJ software and the results were representative of at least four independent experiments.

### Immunofluorescence

To examine the effect of SAG and cyclopamine on the expression and distribution of GLI1, immunofluorescence was performed. FLSs were planted at the density of 2 × 10^4^ cells/mL in glass bottom culture dishes (NEST, San Diego, CA, USA) and treated with SAG or cyclopamine for 48 h. Cells were fixed with 4% paraformaldehyde for 20 min and permeabilized with 0.1% Triton X-100 in phosphate buffered saline (PBS) for 15 min. After blocking at room temperature for 30 min, the cells were incubated with anti-GLI1 antibody (1:200, Abcam) overnight at 4°C. The cells were then incubated with Cy3-conjugated secondary antibody (1:500, Abcam) for 1 h and subsequently stained with DAPI for 15 min. The expression of GLI1 was determined using a confocal fluorescence microscope (Leica Microsystems-TCS SP8, Mannheim, Germany).

### Cell Viability Assay

FLSs were seeded at a density of 3 × 10^4^/mL in 96-well plates. Cells were treated with SAG or SP600125 for 48 h. Cell viability was subsequently assessed using the cell counting kit-8 (CCK-8, Dojindo, Tokyo, Japan) according to the manufacturer's instructions. Cells in the control group were treated with vehicle (DMSO in DMEM supplemented with 10% FBS). The experiments were performed in triplicate and repeated four times.

### Flow Cytometry

For cell cycle phase analysis, FLSs were plated at a density of 5 × 10^4^/mL in 6-well plates and serum-starved for 24 h before incubated with SAG or SP600125. After treated for 18 h, FLSs were harvested and fixed in 70% cold ethanol overnight at −20°C. Fixed cells were subsequently stained using Cell Cycle Analysis Kit (Beyotime, Shanghai, China) according to the manufacturer's instructions. Stained cells were analyzed using a BD LSRFortessa™ Cell Analyzer (Becton Dickinson, San Jose, CA, USA). For each analysis, 10000 events were evaluated with the Flowjo software.

For Ki-67 staining, FLSs were first treated with SAG or SP600125 for 18 h. After being fixed and permeabilized with Foxp3/Transcription Factor Buffer Set (Invitrogen, Carslbad, CA, USA), cells were stained with anti-human Ki-67 PE antibody (1:100, Invitrogen, Carslbad, CA, USA) overnight at 4°C. Stained cells were washed with PBS and analyzed by a BD LSRFortessa™ Cell Analyzer (Becton Dickinson). Ki-67 expression was evaluated using Flowjo software.

For the detection of cell apoptosis, FLSs (5 × 10^4^ cells/mL) were seeded into 6-well plates and treated with SAG or SP600125 for 24 h. Cell apoptosis was determined by AnnexinV/Dead Cell Apoptosis Kit (ESscience, Shanghai, China) according to the manufacturer's protocol. Stained cells were analyzed by a BD LSRFortessa™ Cell Analyzer (Becton Dickinson). AnnexinV-positive/PI-negative staining was regarded as apoptosis and the percentages of apoptotic cells were calculated using Flowjo software. The data were obtained from at least four independent experiments.

### Wound Healing Assay

FLSs were cultured in 24-well plates and wounded with a 200 μL micropipette tip at 90% confluence. After washed three times with serum-free DMEM to remove the debris, the remaining cells were incubated with DMEM containing 2.5% FBS. SAG with or without SP600125 was added to the culture medium. After 12 h of incubation, FLSs were fixed in 4% paraformaldehyde for 15 min and stained with 0.1% crystal violet for 15 min. The number of cells that migrated beyond the scratching line was calculated. The experiments were repeated independently at least four times.

### Cell Chemotaxis and Invasion Assay

The ability of FLS migration was detected using Boyden chambers with 8.0 μm pore size Cell Culture Inserts (Corning Inc., Corning, NY, USA). Briefly, FLSs were trypsinized, collected and re-suspended in serum-free DMEM and planted at a density of 1 × 10^5^ cells/mL in the upper chambers. DMEM containing 10% FBS (600 μL) was used as a chemoattractant. SAG with or without SP600125 was added to the lower compartment after the cells were adherent to the surface. After 16 h of incubation, cells remaining on the upper surface of the filter were removed with a cotton swab. The cells adhering beneath the filter were fixed in 4% paraformaldehyde for 15 min and stained with 0.1% crystal violet for 15 min. Migration ability of FLSs was quantified by calculating the mean number of migrated cells in five random fields at 50 magnifications for each assay.

To examine cell invasion, matrigel-coated membrane was used in the invasion assay. The matrigel basement membrane matrix (Corning Inc., Corning, NY, USA) was diluted with serum-free DMEM and coated on the inserts according to the manufacturer's instructions. FLSs were planted at a density of 2.5 × 10^5^ cells/mL in the upper chambers. The following experiments were similar to the chemotaxis assay. The experiments were performed in duplicate and repeated at least three times.

### Human Active MMP1 Fluorescent Assay

To examine the level of active MMP1 in cell culture supernatants, FLSs were treated with SAG alone or SAG in the presence of SP600125 for 48 h before the cell culture supernatants were collected. The expression of active MMP1 was determined by Fluorokine® E Human Active MMP1 Fluorescent Assay Kit (R&D, MN, USA). All the procedures were performed according to the manufacturer's instructions. The samples were obtained from three independent experiments and assayed in duplicate.

### Enzyme-Linked Immunosorbent Assay (ELISA)

FLSs were treated with SAG alone or in combination with SP600125 for 48 h and human IL-6 (R&D) and IL-8 (Invitrogen, Vienna, Austria) in cell culture supernatants were examined by ELISA Kits. All the procedures were performed according to the manufacturer's instructions.

### Infection of Adeno-Associated Virus (AAV) Overexpressing SHH

AAV overexpressing SHH (CMV-SHH, serotype 5) and blank control virus (CMV-GFP, serotype 5) were purchased from Applied Biological Materials Inc. (Jiangsu, China). FLSs were infected with AAV at a MOI of 10000 and cultured for 7 days before harvested for further experiments. The overexpression of SHH after AAV infection in FLSs was verified by the real time PCR and Western Blot analysis.

### Invasiveness of FLSs *in vivo*

We evaluated the invasiveness of FLSs *in vivo* using a humanized synovitis animal model as previously reported (25, 26). The severe combined immunodeficiency (SCID) mice were employed in the experiment. Briefly, a cartilage-gelatin sponge sandwich was made before implanted subcutaneously into the left side of SCID mouse in the first operation. Two weeks later, cartilage-sponge complex containing FLSs (5 × 10^5^) was inserted under the right flank skin of SCID mouse. FLSs were infected with AAV overexpressing SHH or blank control AAV before implantation. For inhibition of JNK, mice were intraperitoneally injected with SP600125 (15 mg/kg) every 3 days for 45 days. The implants were harvested at day 60 and hematoxylin and eosin (H&E) staining was subsequently performed to evaluate the invasion of FLSs into cartilage. The clinical score including invaded distance and cartilage degradation was assessed by two trained researchers.

### Statistical Analysis

SPSS statistical software, version 20.0, was used for all statistical analyses. Values are presented as Means ± standard deviation (S.D.). Data were obtained from at least 3 independent experiments for the *in vitro* study. For the analysis of protein and mRNA expression, data were normalized and presented as fold change over the controls. The normality of data was examined by Shapiro-Wilk test and homogeneity of variances was examined by Levene test. Comparisons in two groups were performed using independent sample Student's *t*-test. Statistical differences among groups were tested by one-way analysis of variance (ANOVA). *Post hoc* comparisons were made by Dunnett's test. Statistical significance was set at *P* < 0.05.

## Results

### SAG and Cyclopamine Modulate the Activation of SHH Signaling

**Figure 1:**
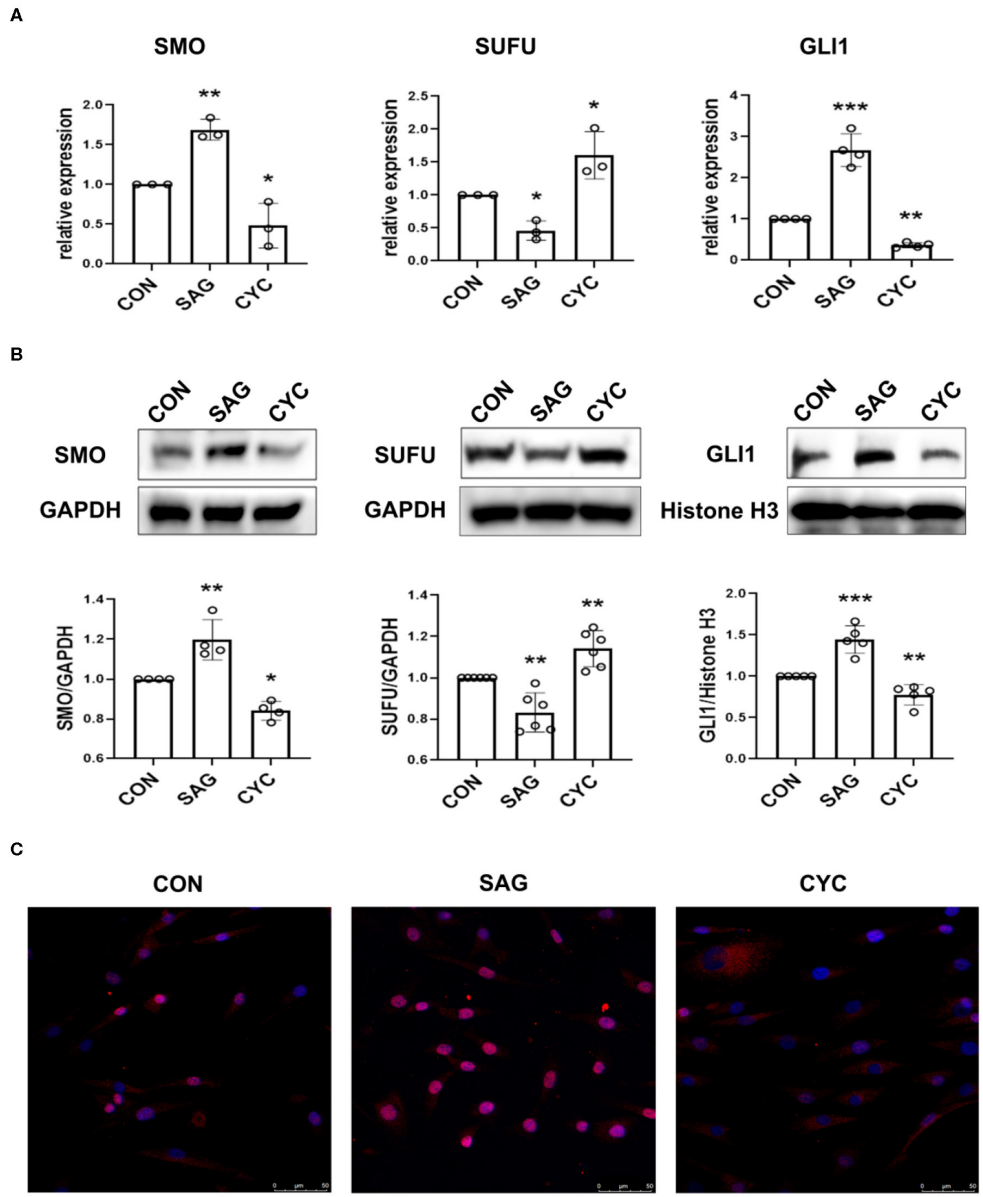
SAG and cyclopamine modulate the activation of SHH signaling. **(A)** SMO, SUFU and GLI1 mRNA expression was evaluated by real-time PCR after FLSs were treated with SAG (1 μM) or cyclopamine (10 μM) for 24 h. Relative quantification of gene expression was performed by the 2^−Δ*ΔCt*^ method. **(B)** The expression of SMO, SUFU and GLI1 was measured by Western Blot after FLSs were treated with SAG (1 μM) or cyclopamine (10 μM) for 48 h. Total protein was isolated for detection of SMO and SUFU and nuclear extracts were prepared for detection of GLI1. Results were normalized to loading control (GAPDH for total protein and Histone H3 for nuclear extracts). The results are shown as the mean ± S.D. **(C)** The distribution of GLI1 protein was measured by immunofluorescence (*n* = 3). FLSs were treated with SAG or cyclopamine for 48 h and stained with anti-GLI1 antibody (red signal) and DAPI (blue signal). Representative images are shown at 200 × magnification. FLSs in control group were treated with vehicle (DMSO supplemented in DMEM with 10% FBS). Data were analyzed using one-way analysis of variance (ANOVA). *post hoc* comparisons were made by Dunnett's test **(A,B)**. *P < 0.05 vs. control group. **P < 0.01 vs. control group. ***P < 0.001 vs. control group. CON, control group; CYC, cyclopamine; SP, SP600125.

SHH signaling is regulated by several key components of the pathway named smoothened (SMO), Suppressor of Fused (SUFU), and GLI zinc-finger proteins. To measure the effects of agonist and antagonist of SHH signaling, SMO, SUFU, and GLI1 expression was determined in the study since GLI1, which is regulated by SMO and SUFU, serves as the transcription activator of SHH signaling and controls the downstream target genes (13). As shown in Figure 1A, treatment of SHH agonist SAG significantly increased the expression of SMO and GLI1 mRNA, while treatment of SHH antagonist cyclopamine reduced the expression of SMO and GLI1 in FLSs. As SUFU is a major negative regulator of SHH signaling, this is expected that SUFU mRNA expression was inhibited by SAG, while the expression of SUFU was promoted by blocking SHH signaling with cyclopamine. The effect of SAG and cyclopamine on the expression of SMO and SUFU protein was similar to the results of mRNA expression (Figure 1B).

To further confirm the effects of SAG and cyclopamine on the activation of SHH signaling, nuclear protein was extracted and the expression of transcription factor GLI1 was determined. In addition, the distribution of GLI1 in FLSs after treated with SAG and cyclopamine was also evaluated in the study. Indeed, we observed that SAG significantly increased the expression of GLI1 protein in the nucleus and inhibition of SHH signaling with cyclopamine decreased the level of GLI1 expression (Figure 1B). The immunofluorescence staining showed that FLSs treated with SAG displayed translocation of GLI1 protein from cytoplasm to nucleus, while cells treated with cyclopamine demonstrated lower proportion of GLI1 accumulation in the nucleus (Figure 1C). These results suggest that SAG and cyclopamine effectively but differently modulate the activation of SHH signaling pathway.

### SHH Agonist and Antagonist Regulate JNK Signaling

**Figure 2:**
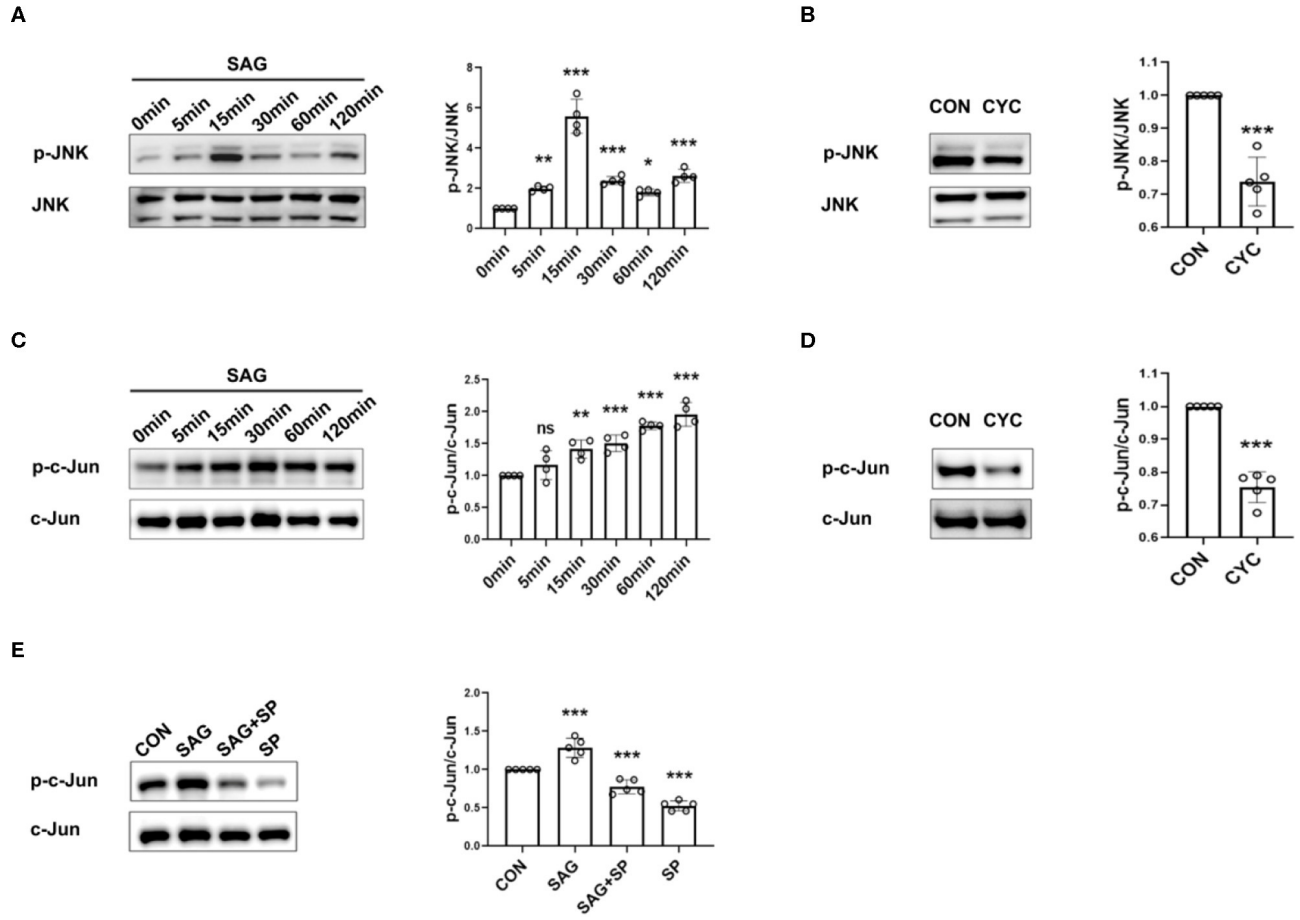
SHH agonist and antagonist regulate JNK signaling. **(A–E)** Effects of SHH agonist (SAG) and antagonist (cyclopamine) on the phosphorylation of JNK and c-Jun were evaluated by Western Blot. FLSs were stimulated with SAG (1 μM) at different time points (5, 15, 30, 60, 120 min) and the levels of phospho-JNK and phospho-c-Jun were determined by Western Blot (*n* = 4) **(A,C)**. To detect the effect of SHH antagonist on JNK signaling, FLSs were treated with cyclopamine (10 μM) for 24 and 48 h before the levels of phospho-JNK **(B)** and phospho-c-Jun **(D)** were detected, respectively (*n* = 5). The expression of phospho-c-Jun was evaluated after FLSs were treated with SAG in the presence of JNK inhibitor SP600125 for 15 min (*n* = 5) **(E)**. Results were normalized to the levels of total JNK or c-Jun. FLSs in control group were treated with vehicle (DMSO supplemented in DMEM with 10%FBS). The results are shown as the mean ± S.D. Data were analyzed using ANOVA with Dunnett's *post hoc* test **(A,C,E)** or independent sample Student's *t*-test **(B,D)**. *P < 0.05 vs. control group. **P < 0.01 vs. control group. ***P < 0.001 vs. control group. ns, non-significant; CON, control group; CYC, cyclopamine; SP, SP600125.

To determine the effects of SAG and cyclopamine on the activation of JNK-MAPK signaling, the phosphorylation of JNK and c-Jun was measured. The results showed that SAG significantly increased the level of phosphorylation of JNK and c-Jun in FLSs, compared with the controls (Figures 2A,C). We also observed that the phosphorylation of JNK and c-Jun was significantly inhibited in the presence of SHH antagonist (Figures 2B,D). Moreover, we also noted that phosphorylation of c-Jun stimulated by SAG was significantly inhibited in the presence of JNK specific inhibitor SP600125 (Figure 2E).

### SHH Agonist Promotes Proliferation of FLSs *via* JNK

**Figure 3:**
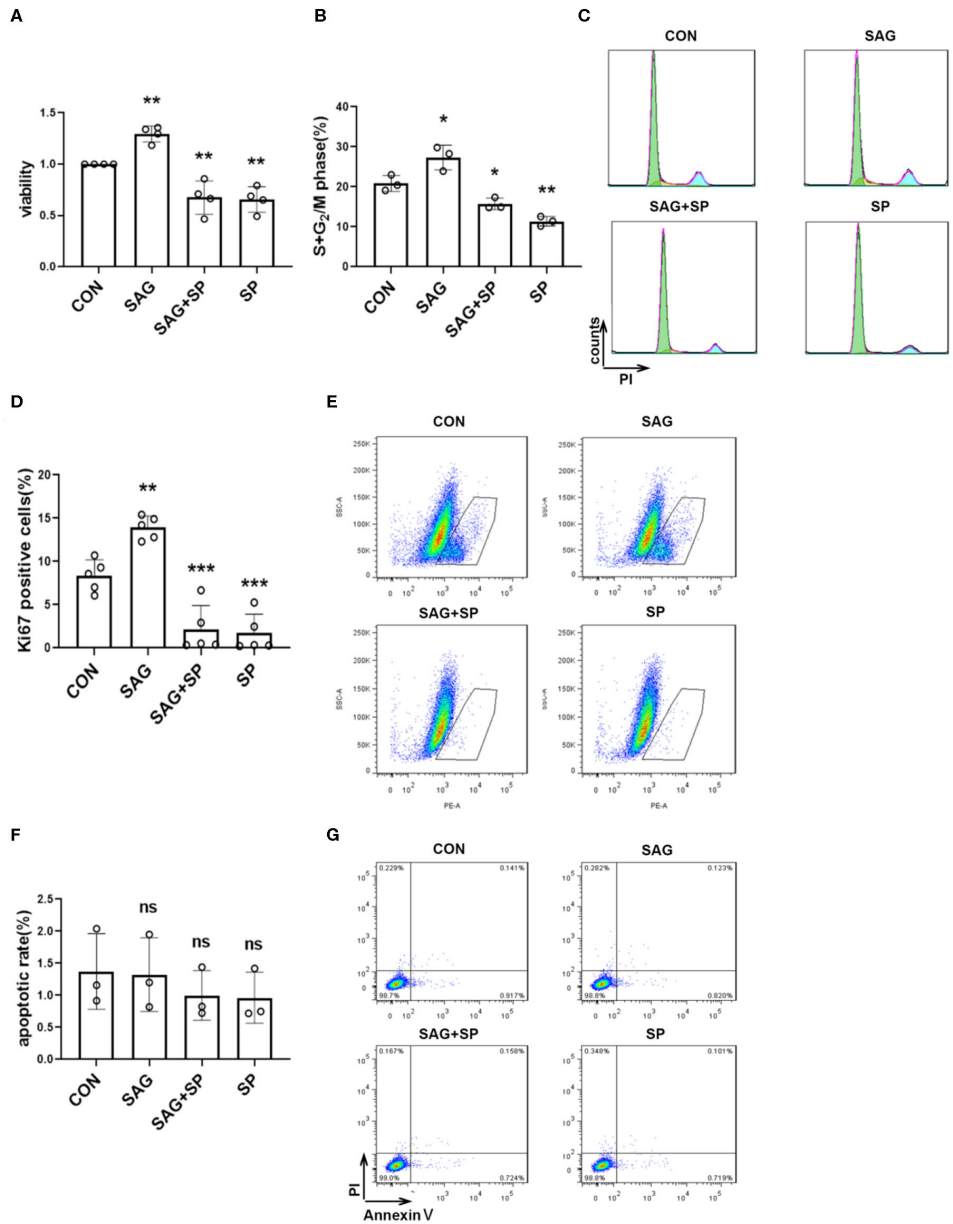
SHH agonist promotes FLSs proliferation *via* JNK. **(A)** FLSs viability was assessed by Cell Counting Kit-8 assay after FLSs were treated with SHH agonist (SAG, 1 μM) alone or SAG with JNK inhibitor (SP600125, 20 μM) for 48 h (*n* = 4). **(B–G)** Cell cycle analysis, Ki-67 expression and cell apoptosis were assessed by flow cytometry. For the detection of cell cycle distribution **(B,C)** and expression of Ki-67 **(D,E)**, FLSs were treated with SAG (1 μM) with or without SP600125 (20 μM) for 18 h. For the detection of cell apoptosis **(F,G)**, FLSs were treated with SAG (1 μM) with or without SP600125 (20 μM) for 24 h. Percentage of apoptosis was defined by the number of AnnexinV^+^PI^−^ cells. FLSs in control group were treated with vehicle (DMSO supplemented in DMEM with 10%FBS). The results are shown as the mean ± S.D. Data were analyzed using ANOVA and *post hoc* comparisons were made by Dunnett's test **(A,B,D,F)**. *P < 0.05 vs. control group. **P < 0.01 vs. control group. ***P < 0.001 vs. control group. ns, non-significant; CON, control group; SP, SP600125.

To elucidate the effects of SHH-JNK signaling on FLSs proliferation, the cell counting kit-8 (CCK-8) assay was performed to evaluate the cell viability. We found that SAG significantly increased cell viability compared with controls (*P* < 0.01). However, the effect of SAG on cell viability was abolished by JNK inhibition (*P* < 0.01, Figure 3A). To exclude the non-specific cytotoxicity of JNK inhibition on cell viability, we further determined the effect of SHH-JNK signaling on cell apoptosis. As shown in Figures 3F,G, SHH-JNK signaling did not affect the cell apoptosis status of FLSs. Therefore, we propose that the effect of SHH-JNK signaling on cell viability is attributed to its impact on cell proliferation.

Additionally, the effect of SHH-JNK signaling on cell cycle distribution was assessed by flow cytometry. The results showed that stimulation of SAG for 18 h significantly increased the percentage of cells in the S and G_2_ phases compared to controls (27.35 ± 3.06% vs. 20.86 ± 1.98%, *P* < 0.05). We also found that percentages of cells in the S and G_2_ phases promoted by SAG were significantly decreased in the presence of SP600125 (15.73 ± 1.42%, Figures 3B,C).

Furthermore, we detected the effect of SHH-JNK on the expression of Ki-67 by flow cytometry. As shown in Figures 3D,E, incubation with SAG for 18 h resulted in an increase of Ki-67 expression in FLSs compared to that of control group (13.92 ± 1.33% vs. 8.34 ± 1.83%, *P* < 0.01). The percentage of Ki-67 positive cells was significantly decreased after incubation with SAG and SP600125 (2.17 ± 2.74%, *P* < 0.001). These results indicate that SHH promotes FLSs proliferation in a JNK-dependent manner.

### SHH Agonist Promotes Migration of FLSs *via* JNK

**Figure 4:**
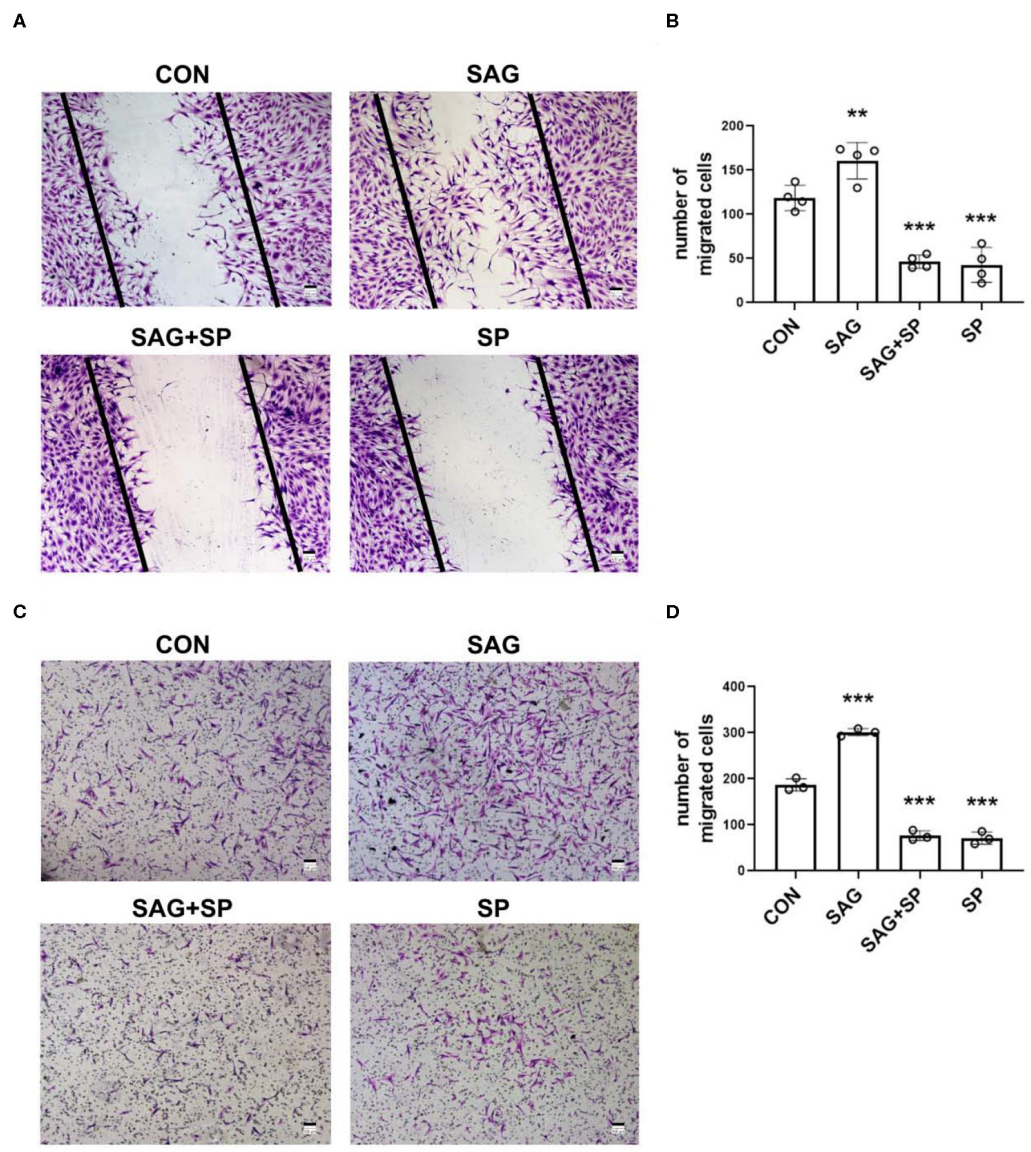
SHH agonist promotes FLSs migration *via* JNK. **(A–D)** FLSs migration was evaluated by wound healing assay and chemotaxis assay. In the wound healing assay, FLSs were treated with SAG (1 μM) or SAG in the presence of SP600125 (20 μM) for 12 h after scratching (*n* = 4) **(A,B)**. In the chemotaxis assay, FLSs were planted in the upper chambers. DMEM containing 10% FBS, SAG (1 μM) with or without SP600125 (20 μM) was added to the lower compartment. Cells were treated for 16 h (*n* = 3) **(C,D)**. The numbers of migrated cells in wound healing assay and chemotaxis assay were calculated. FLSs in control group were treated with vehicle (DMSO supplemented in DMEM with 2.5% FBS in wound healing assay and 10% FBS in chemotaxis assay). The results are shown as the mean ± S.D. Data were analyzed using ANOVA and *post hoc* comparisons were made by Dunnett's test **(B,D)**. **P < 0.01 vs. control group. ***P < 0.001 vs. control group. CON, control group; SP, SP600125.

To examine whether SHH signaling regulates migration of FLSs *via* JNK, we treated FLSs with SAG in the presence of JNK specific inhibitor SP600125 in wound healing and cell chemotaxis assays. We observed that the migration ability of FLSs was significantly enhanced after treatment with SAG, compared with controls (*P* < 0.01). However, we also noticed that the SAG-induced migration ability of FLSs was inhibited by blocking JNK with SP600125 (Figures 4A–D).

### SHH Agonist Increases Invasion of FLSs *via* JNK

**Figure 5:**
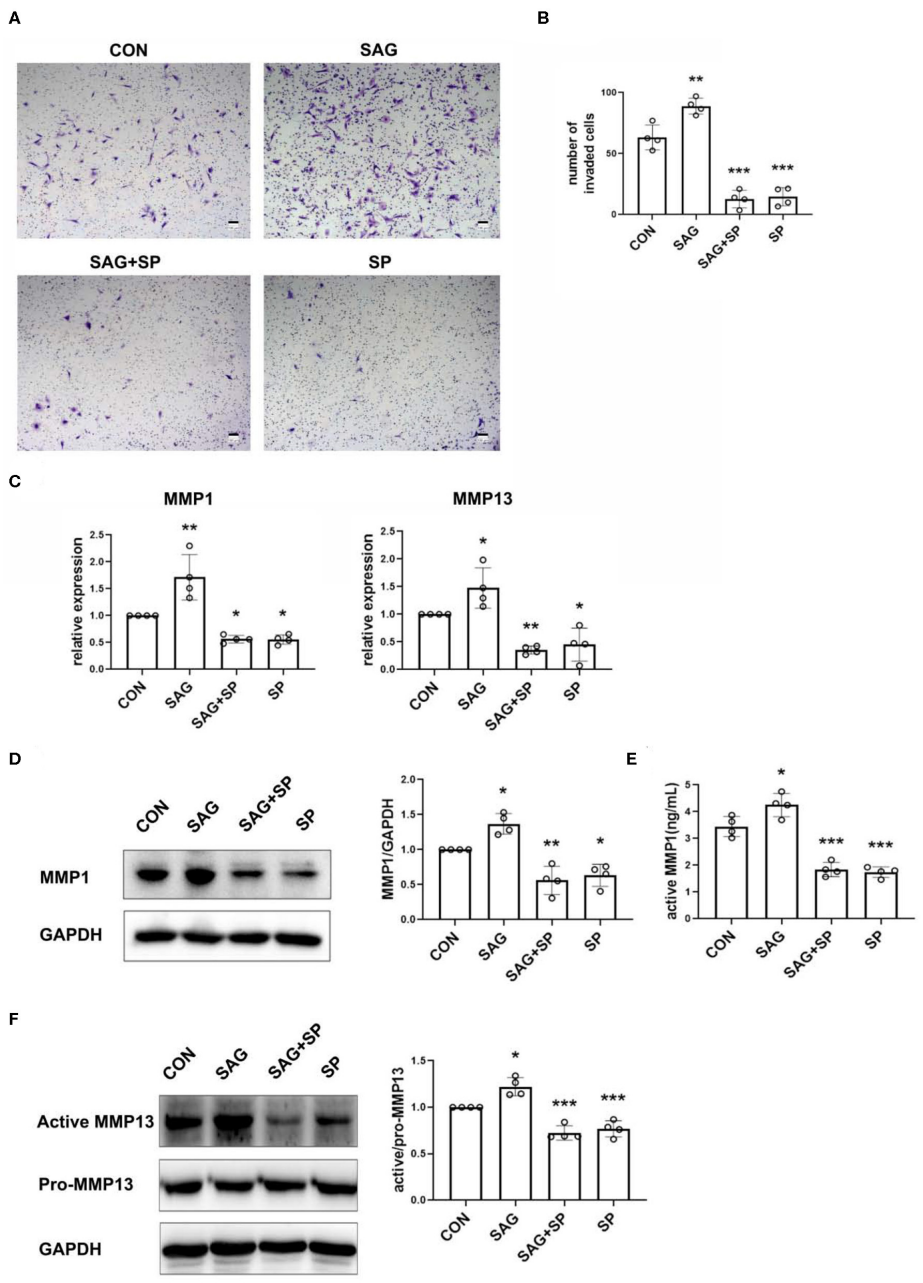
SHH agonist increases FLSs invasion *via* JNK. **(A,B)** FLSs invasion was evaluated by Boyden chambers with matrigel-coated membrane used in the system (*n* = 4). FLSs were planted in the upper chambers. DMEM containing 10% FBS, SAG (1 μM) with or without SP600125 (20 μM) was added to the lower compartment. Cells were treated for 16 h. The numbers of invaded cells were calculated. **(C)** MMP1 and MMP13 mRNA expression was evaluated by real-time PCR after FLSs were treated with SAG (1 μM) or SAG in the presence of SP600125 (20 μM) for 24 h (*n* = 4). **(D,E)** Level of MMP1 protein expression was determined by Western Blot and active MMP1 level in cell culture supernatants was quantified by Fluorokine E activity assay after FLSs were treated with SAG (1 μM) or SAG in the presence of SP600125 (20 μM) for 48 h (*n* = 4). **(F)** The proenzyme and active form of MMP13 were examined by Western Blot (*n* = 4). The results are shown as the mean ± S.D. Data were analyzed using ANOVA and *post hoc* comparisons were made by Dunnett's test **(B–F)**. *P < 0.05 vs. control group. **P < 0.01 vs. control group. ***P < 0.001 vs. control group. CON, control group; SP, SP600125.

As shown in Figure 5A, FLSs stimulated with SHH agonist showed increased invasiveness. To determine whether the SAG-induced increase in cell invasiveness was dependent upon JNK signaling, invasion through matrigel was studied in the presence SP600125 in the lower chambers. The results demonstrated that blockage of JNK with SP600125 significantly decreased the SAG-induced cell invasion (Figures 5A,B).

To further detect the mechanism of SHH-JNK on cell invasion, we examined the levels of MMP1 and MMP13 mRNA and protein expression in FLSs after being treated with SAG alone or SAG in the presence of SP600125. The results showed that mRNA expression of MMP1 and MMP13 was significantly increased in FLSs treated with SAG compared to the controls, and the effect of SAG on MMP1, MMP13 mRNA expression was inhibited in the presence of SP600125 (Figure 5C). In addition, the expression of pro-MMP1 in cell lysates and level of active MMP1 in cell supernatants were also significantly increased by SAG. However, the levels of pro- and active MMP1 were dramatically reduced in the presence of SP600125 (Figures 5D,E). Similarly, SAG also promoted the expression of active MMP13 in FLSs, with an increase in the ratio of active and pro-MMP13. The co-treatment of SAG and SP600125 led to a remarkable decrease in active MMP13 production (Figure 5F).

### SHH Agonist Induces IL-6 and IL-8 Expression in FLSs

To determine the role of SHH-JNK signaling in expression of pro-inflammatory cytokines by FLSs, the mRNA expression of IL-6 and IL-8 was detected after the FLSs were treated with SAG alone or in combination with SP600125. Upregulated expression of IL-6 and IL-8 was observed after stimulation of SAG; however, the elevated expression of IL-6 and IL-8 was not suppressed in the presence of SP600125 (Supplementary Figure 2A). In addition, we detected the production of IL-6 and IL-8 in cell culture supernatants and found that SAG stimulation significantly promoted the expression of IL-6 and IL-8, which was similar to the results of mRNA expression. Of note, we aslo observed that JNK inhibitor did not significantly suppress the production of IL-6 and IL-8 under normal conditions (Supplementary Figure 2B).

### Inhibition of JNK Alleviates FLSs Invasiveness *in vivo* Promoted by SHH

**Figure 6:**
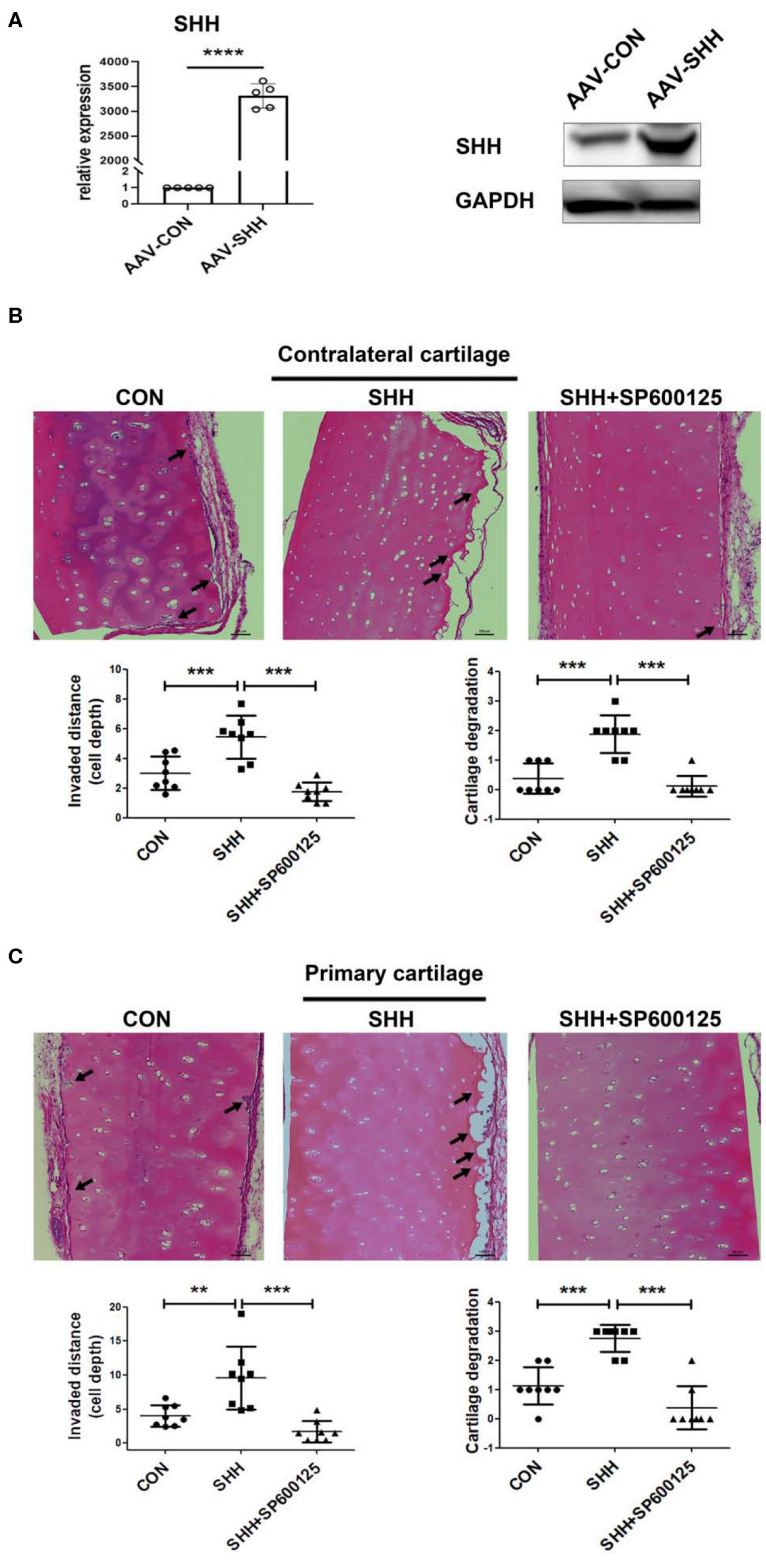
Inhibition of JNK alleviates FLSs invasiveness *in vivo* promoted by SHH. The invasiveness of FLSs *in vivo* was evaluated using a humanized synovitis animal model. SCID mice were used in the experiments (*n* = 5 in each group). A cartilage-sponge complex without FLSs was implanted under the left flank skin of a SCID mouse (contralateral cartilage), and cartilage-sponge complex containing FLSs (5 × 10^5^) was inserted under the right flank skin (primarily cartilage) 2 weeks later. FLSs were infected with AAV virus overexpressing SHH (CMV-SHH, serotype 5) or blank control virus (CMV-GFP, serotype 5) before implantation. The overexpression of SHH in FLSs after AAV virus infection was verified by the real time PCR and Western Blot analysis **(A)**. Mice were injected with SP600125 (15 mg/kg) every 3 days for 45 days. At day 60, the cartilages from mice were harvested and subjected to H&E staining. Representative images were shown at 100 × magnification. The arrow indicated the lesion of cartilage invasion by FLSs **(B,C)**. Data were analyzed using ANOVA and *post hoc* comparisons were made by Dunnett's test. Comparisons in two groups were performed using independent sample Student's *t*-test. **P < 0.01, ***P < 0.001, ****P < 0.0001 vs. SHH group. CON, control group.

To further assess the effect of SHH-JNK signaling on FLSs invasiveness *in vivo*, we employed a humanized synovitis animal model and FLSs were infected with AAV overexpressing SHH before implantation (Figure 6A). As shown in Figures 6B,C, FLSs overexpressing SHH significantly increased the invaded distance and cartilage degradation in the primary and contralateral cartilage, indicating SHH significantly promoted the invasiveness of FLSs. Of note, the destruction of contralateral cartilages which are not directly exposed to FLSs has been attributed to FLSs migration and invasion abilities through the marine body. Therefore, the invasion and destruction of contralateral cartilages not only displayed the invasiveness of FLSs, but also revealed the migration of FLSs *in vivo*. Moreover, we observed that administration of JNK inhibitor to the mice significantly decreased the cartilage invasion by FLSs carrying overexpressed SHH.

## Discussion

Aggressive behavior of FLSs as dysregulated proliferation, increased ability for migration and invasiveness is responsible for pannus formation of RA patients and mediates inflammation and destruction of the joint (27). We have previously reported that SHH signaling promoted FLSs proliferation and migration (19, 20). In line with our findings, a recent study also revealed that inhibition of GLI1 significantly decreased FLSs proliferation (28). However, the mechanism by which SHH signaling promotes the aggressive phenotype of FLSs is not completely clarified.

MAPK cascade is one of the most widely studied signaling pathways in inflammatory diseases. Of the three pathways of classical MAPK family, JNK is a significant contributor to the pathological changes of RA for its ability to phosphorylate c-Jun, which then initiates the expression of MMPs. *In vivo* study showed that inhibition of JNK with antagonist SP600125 alleviated the severity of arthritis and prevented bone destruction in adjuvant-induced arthritis (29). As we previously found that ERK/MAPK was activated by SHH signaling and involved in SHH-driven proliferation and migration of FLSs (23), we are aware that ERK/MAPK is not the only mediator of abnormal behavior of FLSs, and in this study we sought to determine whether JNK contributes to FLSs aggressiveness promoted by SHH signaling.

In the present study, we showed that activation of SHH signaling increased phosphorylation of JNK and c-Jun, conversely, SHH blockade significantly inhibited the activation of JNK signaling. The phosphorylation of c-Jun stimulated by SHH agonist was inhibited in the presence of JNK inhibitor. Additionally, we reported that treating FLSs with SHH agonist significantly increased proliferation, migration, and invasion, and FLSs overexpressing SHH showed increased invasiveness into cartilage *in vivo*, indicating SHH signaling is involved in the activated and aggressive phenotype of FLSs in RA. We further provided evidence that inhibiting JNK with antagonist SP600125 reversed the aggressive behavior of FLSs *in vitro* and *in vivo*. Our observations suggest that SHH signaling promotes FLSs proliferation, migration and invasion in a JNK dependent manner. Although activation of SHH signaling promotes the expression of pro-inflammatory cytokines such as IL-6 and IL-8 in FLSs, the role of JNK signaling in SHH-mediated cytokine expression appears to be not essential.

Previous studies have focused on the crosstalk between SHH-GLI signaling and several oncogenic pathways including MAPKs, PI3K/AKT/mTOR, and TGFβ/SMAD signaling pathways, and implicated the strategy of targeting SHH-GLI and MEK1/2 for the therapy of cancers (30). However, evidence about the interaction between SHH-GLI1 signaling and JNK-MAPK signaling is limited. Previous studies reported that overexpression of SHH elevated phosphorylation of c-Jun in keratinocytes (31). Other studies have shown that JNK activation was involved in bFGF-mediated inhibition of SHH-induced proliferation in granule cell precursors (32). Studies on chemoresistant cancer cells suggested that JNK was activated by SMO through Gβγ and stimulated GLI activity, consequently contributing to chemoresistance (33). Therefore, the mechanism of cross-talk between SHH-GLI and JNK is still unclear and may differ depending on cellular contexts. Our results now for the first time demonstrate that activation of SHH signaling promotes activation of JNK-MAPK and it is likely that JNK is one of the downstream molecules mediating the effect of SHH signaling in FLSs. However, whether JNK activation occurs in upstream of SHH signaling target GLI1 in FLSs remains to be further investigated.

Specifically, MMPs are considered to be major players in destruction of extracellular matrix (ECM) components in RA and other inflammatory diseases (34–37). Additionally, the collagenases MMP1 and MMP13 are associated with the invasiveness of FLSs and articular damage (38, 39). In this study, we have observed SHH signaling significantly increased the expression of MMP1 and MMP13, and blockage of JNK showed a decrease in MMP1 and MMP13 expression. Our study suggests that SHH-JNK signaling may promote invasiveness of FLSs through secretion of MMP1 and MMP13, and could be a potential target to reduce cartilage damage in RA.

## Conclusions

We have identified new evidence implicating the interaction between SHH signaling and JNK-MAPK signaling pathway and its role in the regulation of aggressive phenotype of FLSs from RA patients. These findings suggest that SHH signaling could be a potential therapeutic target to suppress the aggressiveness of RA-FLSs, and prevent or even treat articular damage in RA.

## Data Availability Statement

The raw data supporting the conclusions of this article will be made available by the authors, without undue reservation, to any qualified researcher.

## Ethics Statement

The studies involving human participants were reviewed and approved by Medical Ethics Committee of the Sixth Affiliated Hospital of Sun Yat-sen University. The patients/participants provided their written informed consent to participate in this study. The animal study was reviewed and approved by Guangdong Laboratory Animals Monitoring Institute.

## Author Contributions

JH and SGZ: conceptualization. XF, YC, and FL: formal analysis. SGZ, JH, and SZ: funding acquisition. SZ, YY, YS, and JD: investigation. SZ, YY, YS, and JD: methodology. JH and SGZ: supervision. SZ, YY, and YS: writing—original draft. NO: writing—review & editing. All authors contributed to the article and approved the submitted version.

## Conflict of Interest

The authors declare that the research was conducted in the absence of any commercial or financial relationships that could be construed as a potential conflict of interest.

## Supplementary Material

Supplementary Figure 1 Cell morphology and detection of surface molecules of FLSs. **(A)** FLSs at passage 3 were characterized as spindle-cell morphology under a light microscope (100 × magnification). **(B)** The surface molecules CD90, CD68, CD11b, FcgRII, and FcgRIII receptor were detected by flow cytometry.

Supplementary Figure 2 Effect of SHH-JNK signaling on IL-6 and IL-8 expression in FLSs. **(A)** The mRNA expression of IL-6 and IL-8 was determined by real-time PCR after FLSs were treated with SAG (1 μM) or SAG in the presence of SP600125 (20 μM) for 24 h. **(B)** The expression of IL-6 and IL-8 in cell culture supernatants was measured by an ELISA. The results are shown as the mean ± S.D. Data were analyzed using ANOVA and *post hoc* comparisons were made by Dunnett's test. ^*^*P* < 0.05 vs. control group. ^**^*P* < 0.01 vs. control group. ns, non-significant; CON, control group; SP, SP600125.

## Abbreviations

AAV: adeno-associated virus
ACR: American College of Rheumatology
CCK-8: cell counting kit-8
DMEM: Dulbecco's modified Eagle's medium
DMSO: dimethyl sulfoxide
ECL: enhanced chemiluminescent
ECM: extracellular matrix
ERK: extracellular signal-regulated kinase
FBS: fetal bovine serum
FLSs: fibroblast-like synoviocytes
GLI1: GLI family zinc finger 1
JNK: c-Jun N-terminal kinase
MAPK: mitogen-activated protein kinase
MMP: matrix metalloproteinase
PAGE: polyacrylamide gel electrophoresis
PBS: phosphate buffered saline
PVDF: polyvinylidene fluoride
RA: rheumatoid arthritis
SCID: severe combined immunodeficiency
SHH: sonic hedgehog
SMO: smoothened
SUFU: suppressor of Fused.
