# Supplementary figures and images for: Sonic Hedgehog Regulates Proliferation, Migration and Invasion of Synoviocytes in Rheumatoid Arthritis via JNK Signaling
Source: Front Immunol. 2020 Jun 24;11:1300. doi: 10.3389/fimmu.2020.01300 (PMC7326768; doi:10.3389/fimmu.2020.01300)

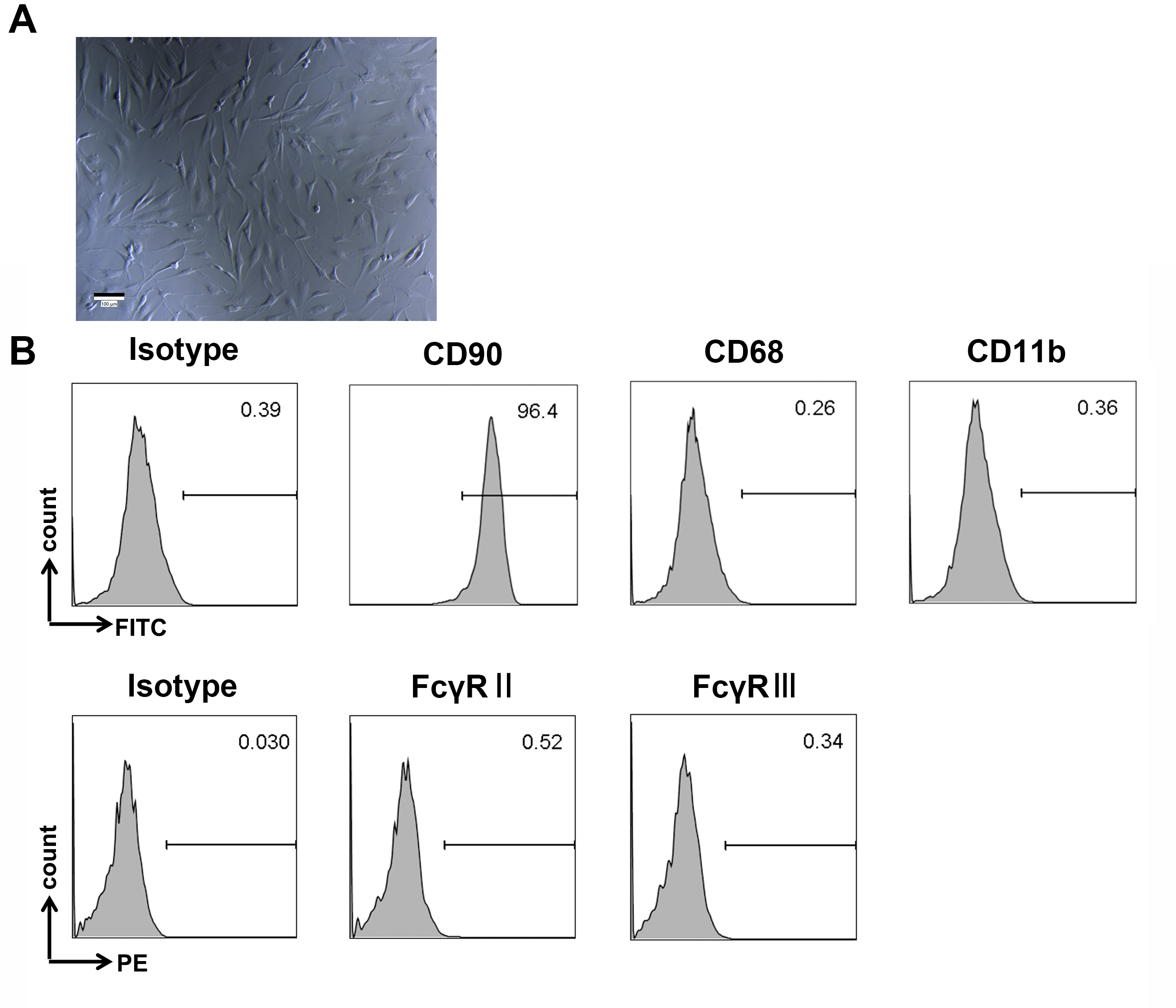

Supplement: Supplementary Figure 1 — Cell morphology and detection of surface molecules of FLSs. (A) FLSs at passage 3 were characterized as spindle-cell morphology under a light microscope (100 × magnification). (B) The surface molecules CD90, CD68, CD11b, FcgRII, and FcgRIII receptor were detected by flow cytometry. [file Image_1.TIF]

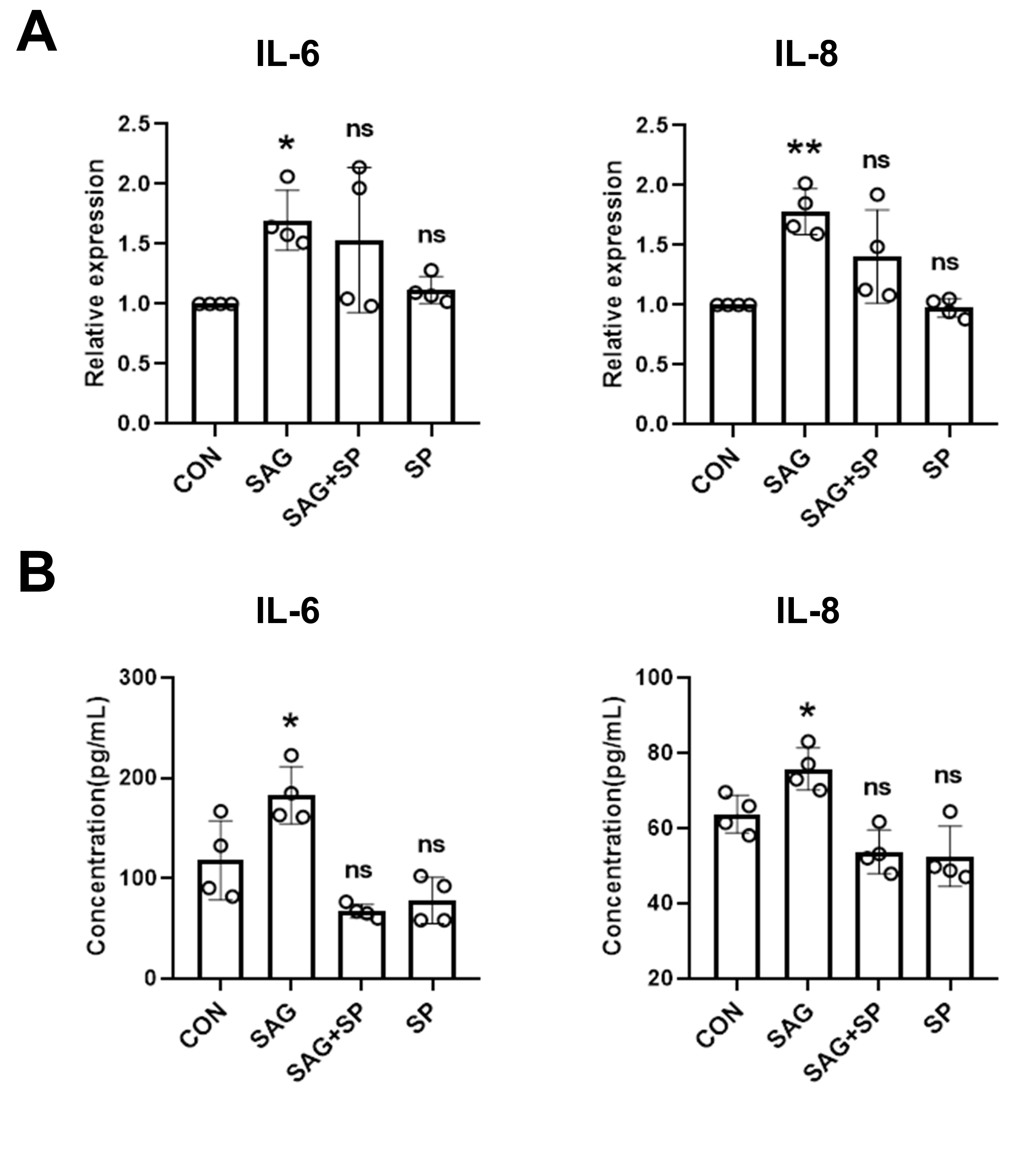

Supplement: Supplementary Figure 2 — Effect of SHH-JNK signaling on IL-6 and IL-8 expression in FLSs. (A) The mRNA expression of IL-6 and IL-8 was determined by real-time PCR after FLSs were treated with SAG (1 μM) or SAG in the presence of SP600125 (20 μM) for 24 h. (B) The expression of IL-6 and IL-8 in cell culture supernatants was measured by an ELISA. The results are shown as the mean ± S.D. Data were analyzed using ANOVA and post hoc comparisons were made by Dunnett's test. *P < 0.05 vs. control group. **P < 0.01 vs. control group. ns, non-significant; CON, control group; SP, SP600125. [file Image_2.TIF]
